# Proteomic analysis of streptomycin resistant and sensitive clinical isolates of *Mycobacterium tuberculosis*

**DOI:** 10.1186/1477-5956-8-59

**Published:** 2010-11-18

**Authors:** Prashant Sharma, Bhavnesh Kumar, Yash Gupta, Neelja Singhal, Vishwa Mohan Katoch, Krishnamurthy Venkatesan, Deepa Bisht

**Affiliations:** 1 Department of Biochemistry, National JALMA Institute for Leprosy and Other Mycobacterial Diseases, Tajganj, Agra, PIN-282001, India; 2 Department of Microbiology & Molecular Biology, National JALMA Institute for Leprosy and Other Mycobacterial Diseases, Tajganj, Agra, PIN-282001, India; 3 Secretary, Department of Health Research, Government of India & Director-General, Indian Council of Medical Research, V. Ramalingaswami Bhawan, Ansari Nagar, New Delhi-110029, India

## Abstract

**Background:**

Streptomycin (SM) is a broad spectrum antibiotic and is an important component of any anti-tuberculosis therapy regimen. Several mechanisms have been proposed to explain the emergence of resistance but still our knowledge is inadequate. Proteins form a very complex network and drugs are countered by their modification/efflux or over expression/modification of targets. As proteins manifest most of the biological processes, these are attractive targets for developing drugs, immunodiagnostics or therapeutics. The aim of present study was to analyze and compare the protein profile of whole cell extracts from *Mycobacterium tuberculosis *clinical isolates susceptible and resistant to SM.

**Results:**

Two-dimensional gel electrophoresis (2DE) and matrix-assisted laser desorption/ionization time-of-flight (MALDI-TOF) mass spectrometry was employed for analyzing the protein profiles. Homology and *in silico *characterization for identified proteins was assessed using BLAST, InterProScan and KEGG database searches. Computational studies on the possible interactions between SM and identified proteins were carried out by a battery of online servers and softwares, namely, CLUSTALW (KEGG), I-TASSER, VMD, PatchDock and FireDock. On comparing 2DE patterns, nine proteins were found consistently overexpressed in SM resistant isolates and were identified as Rv0350, Rv0440, Rv1240, Rv3075c, Rv2971, Rv3028c, Rv2145c, Rv2031c and Rv0569. *In silico *docking analysis showed significant interactions of SM with essential (Rv0350, Rv0440 and Rv2971) and non essential (Rv1240, Rv3075c and Rv2031c) genes.

**Conclusions:**

The computational results suggest high protein binding affinity of SM and suggested many possible interactions between identified proteins and the drug. Bioinformatic analysis proves attributive for analysis of diversity of proteins identified by whole proteome analysis. In-depth study of the these proteins will give an insight into probable sites of drug action other than established primary sites and hence may help in search of novel chemotherapeutic agents at these new sites as inhibitors.

## Background

Tuberculosis is one of the most challenging infectious diseases. Globally, 9.2 million new cases and 1.7 million deaths occur due to this disease [1]. Its impact on public health is further aggravated by co-infection with human immunodeficiency virus, emergence of multi-drug resistant strains and reactivation of the dormant bacteria. Attempt for primary prevention using Bacillus Calmette Guerin (BCG) and other integral vaccines have generally been disappointing though some subunit vaccines are under trial [2]. The excessive emergence of drug resistant tuberculosis has stimulated interest in the understanding of the underlying mechanisms of drug resistance in *Mycobacterium tuberculosis *and significant progress has been made in this field [3]. Streptomycin (SM) is first line anti-tuberculosis drug and preferred for treatment of relapses. It inhibits protein synthesis in susceptible bacteria by interacting with steps of translation. Modification of the primary target of the drug by mutations in the genes encoding either the 16 S rRNA or S12 ribosomal proteins primarily affects the activity of SM and is clinically significant in *Mycobacterium *species [4,5]. However, nearly one third of resistant isolates of *M. tuberculosis *do not have these mutations suggesting the involvement of some other mechanism(s) responsible for resistance [6-8]. Role of efflux pumps in SM resistance have been demonstrated by inhibitor assays [9]. Recently an acetyltransferase gene Rv0262c [10] and mutation within *gidB *gene [11] have also been reported to confer low level SM resistance in *M. tuberculosis*. Still there is a scope for unraveling more underlying mechanisms for SM resistance.

Two-dimension gel electrophoresis (2DE) along with mass spectrometry is a powerful and direct tool to study differential protein expression in cells. Identification and characterization of mycobacterial proteins as drug targets, diagnostics and vaccine candidates have been popular research objectives, but comparative proteome profiling of drug susceptible and resistant isolates remain unexplored in relation to SM. Therefore, the aim of the present study was to compare the protein profiles of cell extracts from *M. tuberculosis *isolates sensitive and resistant to SM.

## Results

**Figure 1:**
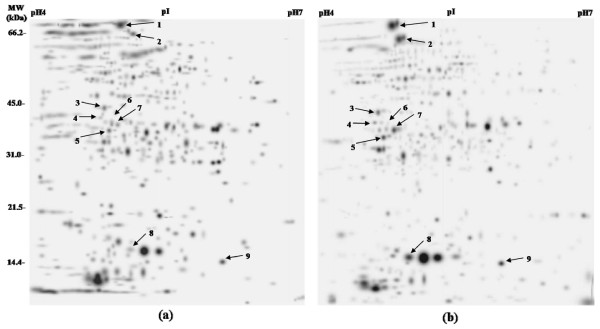
**Composite images of 2 D gels of proteins extracted from *M. tuberculosis *clinical isolates: (a) SM susceptible, (b) SM resistant**. Spots indicated by arrow were excised and analyzed by MS.

**Figure 2:**
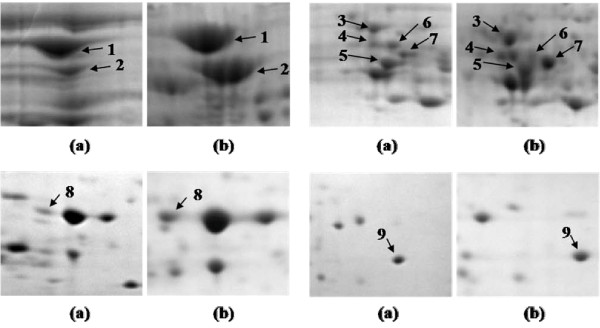
**Magnified regions of 2 D gels showing the overexpressed proteins (a) sensitive isolate; (b) SM resistant isolate**.

This study was focused on the proteins overexpressed in SM resistant *M. tuberculosis *clinical isolates. Protein profiles were compared by 2DE run in triplicates for each isolate. 2DE patterns for SM susceptible and SM resistant isolates are shown as Additional File 1 and Additional File 2 respectively. Spots appearing consistently overexpressed among resistant isolates were further processed. We found nine protein spots to be overexpressed in SM resistant isolates in comparison to sensitive isolates (Figure 1). Magnified regions of these overexpressed proteins are shown in Figure 2. These protein spots were further identified as DnaK, 60 kDa chaperonin2, Malate dehydrogenase, Probable oxidoreductase, Electron transfer flavoprotein subunit alpha, Antigen 84, 14 kDa antigen and two hypothetical proteins by matrix-assisted laser desorption/ionization time-of-flight (MALDI-TOF) mass spectrometry (Table 1) and identity also confirmed by MS/MS (Table 2). The level of over expression has been represented as densitometric ratio in Table 1. All the spots except one exhibited at least two fold over expression. However, Antigen 84 overexpressed maximally more than four fold.

**Table 1:** Details of overexpressed proteins identified by MALDI-TOF mass spectrometry in SM resistant *M. tuberculosis *clinical isolates

Spot No.^†^	**Accession No**.	Proteins identified	Mascot score	Nominal mass (Da)	pI	No. of peptides matched	Sequence coverage (%)	**ORF No**.	Densitometric ratio of protein expression between sensitive vs. resistant isolates
**1**.	P0A5B9 (DNAK_MYCTU)	Chaperone protein dnaK	165	66790	4.85	22	38	Rv0350	1: 1.48
**2**.	P0A520 (CH602_MYCTU)	60 kDa chaperonin 2	85	56692	4.85	12	27	Rv0440	1: 3.17
**3**.	P0A5J6 (MDH_MYCTU)	Malate dehydrogenase	103	34358	4.65	11	51	Rv1240	1: 2.45
**4**.	P95083 (P95083_MYCTU)	Hypothetical protein	98	33194	4.73	9	28	Rv3075c	1: 3.69
**5**.	P95124 (Y2971_MYCTU)	Probable oxidoreductase	97	30516	4.70	11	47	Rv2971	1: 3.10
**6**.	O53275 (ETFA_MYCTU)	Electron transfer flavoprotein subunit alpha (α-ETF)	118	31672	4.71	11	42	Rv3028c	1: 2.97
**7**.	P0A5N2 (AG84_MYCTU)	Antigen 84	132	28260	4.80	9	44	Rv2145c	1: 4.27
**8**.	P0A5B7 (ACR_MYCTU)	14 kDa Antigen (16 kDa Antigen, HSP16.3)	117	16217	5.00	9	70	Rv2031c	1: 2.69
**9**.	O53766 (Y0569_MYCTU)	Hypothetical protein	80	9517	5.70	5	54	Rv0569	1: 2.43

^†^Spot number of protein marked in Figure 1 & Figure 2.

**Table 2:** MALDI-TOF/TOF (MS/MS) analysis of all nine overexpressd proteins in SM resistant *M. tuberculosis *clinical isolates

**Spot No**.	Peak Mass (Da)	Protein Identified	Nominal mass	Mascot Score	pI	Sequence of peptide	**ORF No**.
**1**	1062.611	Chaperone protein dnaK	66790	33	4.85	RTTPSIVAFARN	Rv0350
	1226.711	Chaperone protein dnaK	66790	34	4.85	KDAGQIAGLNVLRI	Rv0350
	1567.931	Chaperone protein dnaK	66790	61	4.85	KLLGSFELTGIPPAPRG	Rv0350
	1645.962	Chaperone protein dnaK	66790	40	4.85	RIVNEPTAAALAYGLDKG	Rv0350
	2613.426	Chaperone protein dnaK	66790	60	4.85	RSETFTTADDNQPSVQIQVYQGERE	Rv0350
**2**	940.555	60 kDa Chaperonin 2	56692	58	4.85	KDLLPLLEKV	Rv0440
	1503.710	60 kDa Chaperonin 2	56692	45	4.85	KGYISGYFVTDPERQ	Rv0440
	1658.900	60 kDa Chaperonin 2	56692	89	4.85	KQIAFNSGLEPGVVAEKV	Rv0440
	1790.868	60 kDa Chaperonin 2	56692	20	4.85	KDETTIVEGAGDTDAIAGRV	Rv0440
	2075.110	60 kDa Chaperonin 2	56692	65	4.85	KTDDVAGDGTTTATVLAQALVRE	Rv0440
	2203.240	60 kDa Chaperonin 2	56692	114	4.85	KKTDDVAGDGTTTATVLAQALVRE	Rv0440
	2317.275	60 kDa Chaperonin 2	56692	62	4.85	KVVVTKDETTIVEGAGDTDAIAGRV	Rv0440
**3**	1693.989	Malate Dehydrogenase	34358	52	4.65	RLASGSLLGPDRPIELRL	Rv1240
	1722.957	Malate Dehydrogenase	34358	38	4.65	KVAVTGAAGQIGYSLLFRL	Rv1240
	1879.944	Malate Dehydrogenase	34358	56	4.65	KGGNWTIVSGLEIDEFSRG	Rv1240
	2423.360	Malate Dehydrogenase	34358	52	4.65	RVGVTGNPANTNALIAMTNAPDIPRE	Rv1240
**4**	995.545	Hypothetical Protein Rv3075c	33194	30	4.73	RLAFGIGDFRR	Rv3075c
	1016.503	Hypothetical Protein Rv3075c	33194	26	4.73	KEFFAEFARD	Rv3075c
	1322.619	Hypothetical Protein Rv3075c	33194	63	4.73	RWFGDGNADWVRI	Rv3075c
	1491.919	Hypothetical Protein Rv3075c	33194	32	4.73	RLPNVPIVALVETARG	Rv3075c
	1583.780	Hypothetical Protein Rv3075c	33194	64	4.73	RDTGFGEDPATLAYARS	Rv3075c
	1648.036	Hypothetical Protein Rv3075c	33194	45	4.73	KRLPNVPIVALVETARG	Rv3075c
**5**	897.627	Probable Oxidoreductase	30516	15	4.70	KTPAQVLLRW	Rv2971
	1105.658	Probable Oxidoreductase	30516	27	4.70	KLATPDQGFTRS	Rv2971
	1368.911	Probable Oxidoreductase	30516	72	4.70	RWNLQLGNAVVVRS	Rv2971
	1382.701	Probable Oxidoreductase	30516	12	4.70	RWNLQLGNAVVVRS	Rv2971
**6**	1069.609	Electron transfer flavoprotein subunit alpha	31672	43	4.71	KVAPQLTEAIKA	Rv3028c
	1341.748	Electron transfer flavoprotein subunit alpha	31672	56	4.71	RIGSGLLVDVVDVRE	Rv3028c
	1577.902	Electron transfer flavoprotein subunit alpha	31672	113	4.71	MAEVLVLVEHAEGALKK	Rv3028c
	1706.093	Electron transfer flavoprotein subunit alpha	31672	65	4.71	MAEVLVLVEHAEGALKKV	Rv3028c
	1973.063	Electron transfer flavoprotein subunit alpha	31672	69	4.71	RAAVDSGYYPGQFQVGQTGKT	Rv3028c
	2024.322	Electron transfer flavoprotein subunit alpha	31672	67	4.71	KTVSPQLYIALGISGAIQHRA	Rv3028c
	2692.697	Electron transfer flavoprotein subunit alpha	31672	14	4.71	KNGLVLVIDGQLWTITEFQHVKPGKG	Rv3028c
**7**	1088.702	Antigen 84	28260	22	4.80	RLIEENSDLRQ	Rv2145c
	1171.748	Antigen 84	28260	32	4.80	RANAEQILGEARH	Rv2145c
	1807.228	Antigen 84	28260	38	4.80	RLKTYLESQLEELGQRG	Rv2145c
	1817.241	Antigen 84	28260	33	4.80	RVLSLAQDTADRLTNTAKA	Rv2145c
**8**	885.507	14 kDa antigen	16217	21	5.00	MATTLPVQRH	Rv2031c
	1162.563	14 kDa antigen	16217	47	5.00	RSEFAYGSFVRT	Rv2031c
	1715.053	14 kDa antigen	16217	28	5.00	KGILTVSVAVSEGKPTEKH	Rv2031c
	1869.098	14 kDa antigen	16217	46	5.00	RAELPGVDPDKDVDIMVRD	Rv2031c
**9**	929.516	Hypothetical protein Rv0569	9517	22	5.70	KVGDWLVIKG	Rv0569
	1109.493	Hypothetical protein Rv0569	9517	12	5.70	KGATIDQPDHRG	Rv0569
	1163.530	Hypothetical protein Rv0569	9517	4	5.70	RSSDGSPPYVVRW	Rv0569
	1269.646	Hypothetical protein Rv0569	9517	17	5.70	RFGAVQSAILHARG	Rv0569

Results of computational analysis of all nine overexpressed proteins using different softwares and servers are as follows.

### BLAST Analysis

Blastp analysis was carried out for two hypothetical proteins and two proteins with unknown functions. Hypothetical protein (Rv3075c) was found highly conserved (99 ± 0.05% identical) in few species of mycobacteria and in others it acts as citrate lyase subunit beta-like protein (CitE) or HpcH/HpaI aldolase. Other one hypothetical protein (Rv0569) was also found highly conserved in mycobacteria and other microbes and significant function could not be assigned except in few microbes as signal-transduction protein (*Frankia *and *Nocardioides sps*.) and DNA-binding protein (*Streptomyces coelicolor *&*S. lividans*). Probable oxidoreductase (Rv2971) appeared highly conserved in some species of mycobacteria, some as 2, 5-diketo-D-gluconic acid reductase A (*M. avium*) or morphine 6-dehydrogenase (*M. smegmatis*). Probable electron transfer flavoprotein alpha-subunit (fixb) was also highly conserved in mycobacteria and other microbes.

### Phylogenetic analysis

CLUSTALW analysis was performed for all overexpressed proteins. Rv0350, Rv0440 and Rv1240 were highly conserved among all organisms and Rv0350 had 51% homology with human heat shock 70 kDa protein, Rv0440 showed 45% homology with human heat shock 60 kDa protein-1 and Rv1240 exhibited 48.93% homology with human malate dehydrogenase. Rv3075c had 20.19% homology with CitE of human, 86.31% with *M. marinum *& 85.66% with *M. ulcerans*, Rv2971 revealed 34% homology with human aldo-keto reductase and Rv3028c had 39% homology with human electron transfer flavoprotein. Rv2145c had 18.34% homology with ankyrin repeat domain 24 protein of human. Rv2031c had 29.86% homology with HSP20/alpha crystallin family protein of *M. avium *and 20% homology with 18 kDa antigen of *M. leprae*. However it showed 17% homology with human outer dense fiber of sperm tails protein. The hypothetical protein (Rv0569) did not show homology with any human protein.

### InterProScan analysis

**Figure 3:**
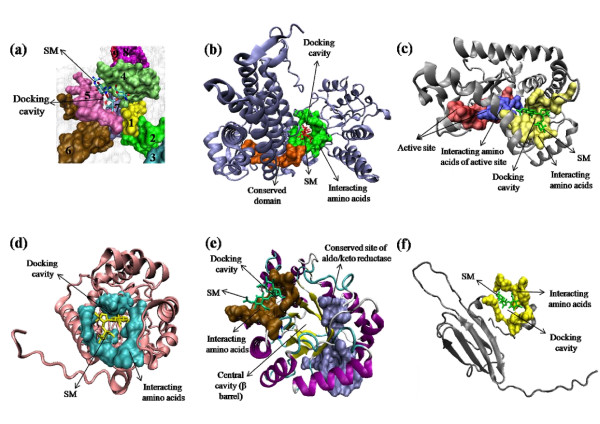
**3D models of overexpressed proteins showing docking with SM**. Residues constituting interacting site, active site and conserved site are represented as space filled models with rest of the structure represented by cartoon structures. **(a) **Rv0350: Nine motifs are marked by numerals, docking cavity and SM are indicated by arrows and motifs 4, 5 & 7 are interacting with SM. **(b) **Rv0440: Red coloured SM, green coloured cavity and orange coloured conserved domain has been marked. Conserved domain is in the close vicinity of interacting site. **(c) **Rv1240: SM (green) is interacting with active site residues (blue) and other residues in the close vicinity (yellow) in the cavity. **(d) **Rv3075c: SM (yellow) interacting clearly with the central cavity residues (blue) of the globular protein. **(e) **Rv2971: SM (green) binding at the opposite side (brown) from conserved site of aldo/keto reductase (light blue) in the protein. Central cavity is present in the middle of complete β-barrel. **(f) **Rv2031c: SM (green) interacting with the outer part (yellow) of the protein in place of conserved HSP20-like chaperone domain.

**Figure 4:**
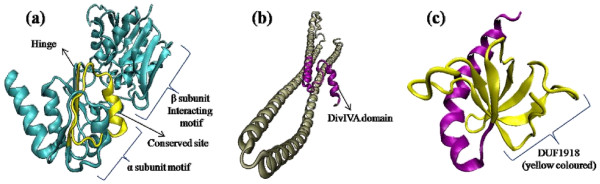
**3D models of proteins found non-interacting with SM represented by cartoon structures**. **(a) **Rv3028c: Protein contains one α-subunit motif and one β-subunit interacting motif and one conserved site in the α-subunit motif. **(b) **Rv2145c: Whole protein consists of only α-helix and contains DivIVA domain indicating its role in cell division/cell shape. **(c) **Rv0569: Domain of unknown function (DUF1918) is present in the β-barrel of protein.

InterProScan analysis of Rv0350 showed nine signature motifs [SPRINT: PR00301] (Figure 3a) and motifs 1, 5 and 6 were most conserved [INTERPRO: IPR018181]. One peptide binding domain [SUPERFAMILY: SSF100920] and two actin like ATPas domains were also present in it [SUPERFAMILY: SSF53067]. Rv0440 showed five conserved motifs [SPRINT: PR00298], one conserved site (from residues 403-414) for 60 kDa Chaperonin [INTERPRO: IPR018370] (Figure 3b) and GroEL apical domain like region from residues 182-374 [SUPERFAMILY: SSF52029]. Rv1240 revealed one characteristic motif which provides a signature for L-lactate/malate dehydrogenase [PFAM: PF00056], one active site from residues 156-168 [PROSITE: PS00068] (Figure 3c) and NAD (P)-binding Rossmann fold domains [SUPERFAMILY: SSF51735]. Rv3075c showed characteristic HpcH-HpaI motifs [PFAM: PF03328], phosphoenolpyruvate/pyruvate domain from residues 22 to 239 [SUPERFAMILY: SSF51621] and citrate lyase beta subunit domain from residues 1-288 which provides a signature for lyase/aldolase activity [UNIPROT: PIRSF015582]. Rv2971 showed five Aldo/keto reductase subgroups 43-67, 99-117, 131-148, 165-194 and 202-226 [SPRINT: PR00069] out of which one is conserved site of aldo/keto reductase from residues 131-148 [PROSITE: PS00062] (Figure 3e). Rv3028c showed alpha/beta-subunit motif from residues 4-125 [PFAM: PF01012], one alpha subunit from residues 197-227 [PFAM: PF00766] and one conserved site found from residues 257-283 in alpha subunit at C-terminal side [PROSITE: PS00696] (Figure 4a). Rv2145c showed divIVA motif from residues 3-61 [PFAM: PF05103] and DivIVA domain from residues 3-39 [CMR: TIGR03544] (Figure 4b). Rv2031c confirmed its relation with Hsp20/alpha crystallin family [PFAM: PF00011], heat shock hsp20 proteins family profile [PROSITE: PS01031] and HSP20-like chaperone [SUPERFAMILY: SSF49764]. Amino acid residues were almost common for showing its relation with all three families. Signature for chaperone proteins and HSP20 family motif is also present in the same region from residues 22-142 [PANTHER: PTHR11527]. Rv0569 showed domain of unknown function DUF1918 from residues 1-58 [PFAM: PF08940] (Figure 4c). None of the nine proteins had signal peptide or transmembrane domains neither they had any site for post translational modifications.

### 3D modeling and docking

All selected 3D models showed less than 2% discrepancy from Ramachandran plot. These models were further explored for *in silico *docking studies to identify the binding of SM. Parameters used for selection of 3D models and their docking with SM are represented in Table 3. There were possible active cavities observed on the surface of the 3D structures. Molecular docking of SM with secondary structures of Rv0350, Rv0440, Rv1240 & Rv3075c proteins showed successful binding (Figure 3) into the central cavity of the protein and the drug molecule fits well in the cavity. However, with Rv0350 and Rv3075c, drug showed binding at the conserved active site whereas in Rv1240, SM is interacting with four residues of conserved active site and for Rv0440 drug showed binding in the close vicinity of the conserved site. In docked complex of Rv2971 and Rv2031c, binding of drug was far from the conserved/active site (Figure 3). Remaining three proteins Rv3028c, Rv2145c & Rv0569 did not show significant binding of the drug and their 3D models are shown in Figure 4.

**Table 3:** 3D modeling and docking parameters used for bioinformatic analysis

Identified protein's ORF number	TM-score	RMSD value (Å)	Global energy	Attractive Vander-wall force	Repulsive Vander-wall force	ACE	Interacting amino acid residues	Remarks
**Rv0350**	0.75 ± 0.10	7.2 ± 4.2	-45.45,	-24.49,	5.13	-9.21	11, 70, 71, 119, 121, 122, 126, 175, 177, 193-195 and 197	Interacting amino acids belong to or are in close proximity of signature motifs 1, 4 and 5
**Rv0440**	0.44 ± 0.08	5.4 ± 3.4	-42.74,	-25.89,	5.64	-5.26	117, 172-175, 192, 206, 216, 212, 320, 322, 324, 326, 327, 329, 391, 395, 398, 399 and 402	Interacting residues are in the close vicinity of the one conserved site
**Rv1240**	0.94 ± 0.05	3.2 ± 2.2	-51.86,	-22.83,	5.69	-15.08	156-168	Amino acid residues found interacting with SM are from the active site for function
**Rv3075c**	0.49 ± 0.15	10.5 ± 4.6	-44.95,	-21.94,	5.20	-10.62	31, 57, 159-162, 165, 198, 200, 224, 226, 278, 281, 282, 285 and 286	amino acid residues found interacting with SM are situated at the central cavity of the molecule
**Rv2971**	0.93 ± 0.06	2.9 ± 2.1	-41.57,	-18.69,	6.30	-14.35	30, 31, 195-200, 237-340 and 243	amino acid residues found interacting with SM are situated far from the conserved site (residue 131 to 148)
**Rv3028c**	0.99 ± 0.04	2.4 ± 1.8	-39.04,	-21.43,	8.55	-10.03	poor docking	-
**Rv2145c**	0.30 ± 0.09	15.4 ± 3.4	-35.93	-22.04	22.52	-13.70	poor docking	-
**Rv2031c**	0.82 ± 0.09	3.2 ± 2.3	-41.10,	-26.91,	19.30	-14.57	8, 10, 14, 18, 21, 22, 25, 28, 32 and 34	SM interacts with region exclusively conserved in genus *Mycobacterium*, no active site found
**Rv0569**	0.83 ± 0.08	2.1 ± 1.7	-30.57,	-17.34,	7.52	-12.89	poor docking	-

## Discussion

The 2DE profile of SM resistant *M. tuberculosis *clinical isolates exhibited many proteins that were overexpressed. Protein spots 1 and 2 are chaperone protein 70 kDa DnaK (Rv0350) and 60 kDa Chaperonin-2/GroEL-2 (Rv0440) respectively. These essential genes prevent misfolding and promote the refolding and proper assembly of unfolded/misfolded polypeptides generated under stress conditions. Rv0350 have characteristic peptide binding domain and ATPase domain, which indicate its role in active protein refolding and proper assembly. The most conserved motifs are interacting with docked SM which indicates that the drug may inhibit/modulate the functioning of this protein and might influence SM toxicity. In prokaryotes DnaK is known to negatively regulate/auto-regulate expression of many HSP proteins by promoting degradation of σ^32 ^heat shock transcriptional regulator [12] and σ^32 ^regulates over expression of other HSPs [13]. Thus blocking of DnaK activity not only positively regulates its expression but may also change HSP profile of the cell. Rv0350 has also been found overexpressed in SM induced culture of SM mono-resistant clinical isolate of *M. tuberculosis *[14]. Rv0440 (GroEL) is the second copy of 60 kDa Chaperonin in *M.tuberculosis *genome. This gene is situated away from classic GroEL-Cpn10 operon and suggests a specialised regulation in *M.tuberculosis*. SM showed binding in the close vicinity of the conserved site on the apical domain, which is a peptide binding domain [15] and therefore it may be predicted that it could be a possible inhibitor/modulator. Duplicate copies of this gene suggest its importance as an energy independent chaperonin in slow metabolism efficient genome of *M. tuberculosis *[16]. Over expression of this gene could be to compensate inhibited/modulated molecules or to neutralise drug by binding. Further, spot 8 corresponding to Rv2031c encodes 14 kDa antigen (HSP16.3/HSPX) which is a member of the small heat-shock protein family of chaperones. It has been shown to be induced under oxygen-deficient conditions [17]. Its role in maintenance of long term viability during latent, asymptomatic infections and in replication during initial infection has also been proposed. Docking studies revealed that the SM binds to a demarcated cavity comprising of 10 amino acids though the interacting amino acids do not belong to any known activity region but these are highly conserved among mycobacteria. These leads can be exploited for better understanding of function of these domains by employing inhibition studies.

Spot 3 (Rv1240) encodes malate dehydrogenase, which is involved in the conversion of malate to oxaloacetate. Role of this protein in *M. tuberculosis *drug resistance remains to be elucidated. SM interacts with the amino acids of conserved active site of Rv1240, which suggests that the drug can affect the activity of this enzyme. Spot 5 (Rv2971) belonging to oxidoreductase of aldo/keto reductase family is probably involved in cellular metabolism. Previous studies also found that this protein was differentially expressed between BCG and H37Rv and was regarded as a candidate antigen for development of novel vaccine [18,19]. Furthermore, this protein has also been reported to be differentially expressed between isoniazid (INH) susceptible and resistant strains [20]. Residues found interacting with SM were situated far from conserved site and therefore it is suggested that this protein might be playing a secondary role in imparting resistance.

Spot 6 (Rv3028c) is an electron transfer flavoprotein alpha subunit. Bioinformatic analysis indicated that alpha subunit provides a signature for the electron transfer flavoprotein family. The electron transfer flavoprotein serves as a specific electron acceptor for other dehydrogenases. It transfers the electrons to the main respiratory chain via ETF-ubiquinone oxidoreductase (ETF dehydrogenase) [21] and participates in the oxidation of fatty acids [22]. The 3D structure of Rv3028c adopts a typical bi-lobed structure with four alpha helices each packing against the hydrophobic beta sheet comprising of 5 plates in alpha subunit motif and 7 plates in beta subunit interacting motif. No significant *in silico *interactions with SM were found. Further studies regarding substrate specificity & interacting proteins might reveal a relation with SM resistance. Spot 7 (Rv2145c) encodes antigen 84 and also corresponds to wag31 which was originally identified as antigen of pathogenic mycobacteria that is recognized by serum from tuberculosis patients [23]. Studies indicate that this gene is a homologue of the cell shape/cell division protein DivIVA and one of the substrates of PknA and PknB [24]. Orthologs of Rv2145c have also been found as immunogenic, cell division initiation protein or secreted antigen Wag31 in some microbes. Rv3028c & Rv2145c were also found overexpressed in INH resistant *M. tuberculosis *isolates [20]. Its 3D structure showed that the protein has a bi-lobbed ribbon structure comprising mostly of alpha helices & the conserved site of the protein had no evident interaction with SM.

Two protein spots 4 (Rv3075c) and 9 (Rv0569) which encode hypothetical protein could not be assigned any function. Phylogenetic analysis of these two proteins revealed existence of their homologous sequences in other mycobacterial species with known as well as unknown functions and reflected dynamic and interesting scenarios of evolutionary importance. Rv3075c, besides showing homology with hypothetical protein, also showed homology with CitE of some mycobacterial species. While the bacterial citrate lyase is a heterotrimeric complex with three subunits, the *M. tuberculosis *genome does not contain α and γ subunits of this complex, implying that *M. tuberculosis *CitE act differently from other bacterial CitE proteins [25]. These data hint that the biochemical function of the *M. tuberculosis *and human CitE may differ from other bacterial CitE proteins, and that *M. tuberculosis *CitE may be critical for pathogenesis, encompassing part of a novel pathway for fatty acid biosynthesis or anaerobic energy metabolism [26].

SM docking with Rv3075c revealed that the drug binds to the active site of the protein and interacts with 16 residues, out of which, one residue (162, Asp) has been reported as a part of active site in *E. coli *[27,28]. Thus it is suggested that the SM is binding at the active site of Rv3075c. It is assumed that the drug might interfere with protein function. blastp analysis of the other hypothetical protein (Rv0569) did not show any significant identity (exhibited homology with hypothetical proteins). Phylogenetic and blastp analysis exhibited that Rv0569 might work as signal transduction protein or DNA binding protein but no function has been assigned. 3D model of this protein showed semi-lunar beta barrel with a very compact structure and an alpha helix protruding out instead of packing against hydrophobic beta plates. Rv0569 showed no significant binding with SM. It is quite likely that being a possible signaling protein it might modulate the expression of other proteins.

## Conclusions

To conclude, this study has employed proteomic approach, which is a direct method, to identify proteins from resistant *M. tuberculosis *isolates compared to sensitive isolates. Nine protein spots were consistently overexpressed in SM resistant isolates. We expect that these proteins might be contributing in conferring resistant phenotype to the isolates. Further these proteins were subjected to advanced bioinformatics analysis to generate an understanding of the subtle relation of SM with the overexpressed proteins. Homology searches and InterProScan generated insights to the possible functions and essential domains of the proteins. Rv0350, Rv0440, Rv1240, Rv3075c, Rv2971 and Rv2031c showed significant interaction *in silico *with SM thus their over expression in the resistant isolates could be compensating the inhibited/modulated molecules. Other proteins which are overexpressed but do not exhibit good binding with drug might be indirectly associated with SM. The elucidated mechanisms and associations may be further exploited to develop newer therapeutic agents derived from SM.

## Methods

### Mycobacterial growth and drug susceptibility testing

Six SM resistant and three sensitive (to five first line drugs) *M. tuberculosis *clinical isolates were obtained from Mycobacterial Repository Centre of our Institute. Susceptibility testing was performed by conventional LJ proportion method [29]. Bacteria were grown in Sauton's liquid medium at 37°C for four weeks (late log phase).

### Preparation of mycobacterial cell extract

Mycobacterial cell extract was prepared according to modified protocol of Brodie et al., [30]. Cells were washed three times with normal saline and then suspended in sonication buffer (50 mM Tris-HCl containing 10 mM MgCl_2_, 0.1% sodium azide, 1 mM PMSF and 1 mM EGTA; pH 7.4) at a concentration of 1 g wet cell mass per 5 ml and then broken by intermittent sonication for 15 min at 4°C using sonicator (Sonics & Materials Inc, Newtown, CT, USA). The homogenate was centrifuged at 12,000 g for 20 min at 4°C. Pellets were discarded and supernatant was stored at -70°C until used.

### Protein precipitation with SDS-TCA-acetone

Cell extracts were treated with 1% SDS and then subjected to trichloro acetic acid (TCA)-acetone precipitation procedure [31]. 10% TCA was added to the cell extract, the mixture was incubated at -20°C overnight and then precipitated protein was collected by centrifugation (18,000 g, 4°C, 15 min). It was again washed twice with 100% ice cold acetone and allowed to air dry. The protein pellet was suspended in appropriate volume of two-dimensional rehydration buffer (BIO-RAD, Hercules, CA, USA). Protein concentration was estimated using the Bradford assay [32].

### Two-dimensional gel electrophoresis (2DE)

Isoelectric focusing (IEF) was carried out using the method of 'in gel rehydration' [33] with slight modifications as described previously [34]. Immobilized pH gradient (IPG) strips of pH 4-7 and length 17 cm (BIO-RAD, Hercules, CA, USA) were rehydrated overnight at 20°C with 500 μg protein which was mixed with rehydration buffer. Strips were then focused on an IEF unit PROTEAN IEF Cell (BIO-RAD, Hercules, CA, USA) at 20°C using the following four step program: a) 0-250 V for 2 h in linear mode; b) 250 V constant for 2 h in rapid mode; c) 250-5000 V for 4 h in linear mode; and d) 5000 V constant until 35 kVh reached. The current limit was set at 50 μA per strip. After IEF, IPG strips were equilibrated for 15 min in equilibration buffer I (6 M urea, 2% SDS, 0.375 M Tris; pH 8.8, 20% glycerol) containing 130 mM dithiothreitol (DTT) followed by equilibration buffer II containing 135 mM iodoacetamide instead of DTT for 15 min.

Proteins were separated in second dimension on 12% SDS-PAGE [35] in a vertical electrophoretic dual gel unit PROTEAN II XI (BIO-RAD, Hercules, CA, USA) at constant voltage of 250 V for 5-6 h and gels were stained with coomassie brilliant blue R250 to visualize proteins. Images of gels were acquired by Chemidoc (BIO-RAD, Segrate [Milan], Italy) using Quantity One software (BIO-RAD, Hercules, CA, USA). 2 D gels were analysed using PDQuest Advanced software (version 8.0) (Bio-Rad, Hercules, CA, USA). Images were analysed using stepwise spot detection and spot matching followed by differential expression analysis. PDQuest employs Student t-test and enumerates spots with differential intensity of significant levels. Resultant composite images for susceptible and resistant isolates were manually checked for artifactual spots, merged spots, and missed spots (Figure 1) and few spots with more isolate specific variability were omitted in the down stream processing. Equal amount of protein was loaded in all gels and experiments were repeated at least three times.

### *In-gel *digestion with trypsin

Method of Shevchenko et al., [36] was followed with slight modifications as described earlier [34]. Protein spots of interest were excised from gels using spot picker 'Investigator ProPic' (Genomic Solutions, Huntingdon, UK) and collected in 96 well PCR plate. Digestion of proteins and spotting of peptides on MALDI-TOF target plate was carried out using protein digester 'Investigator ProPrep' (Genomic Solutions, Huntingdon, UK). The gel plugs were destained and dehydrated by washing three times (~10 min) with 25 mM NH_4_HCO_3_-50% acetonitrile (ACN) (1:1). Dried gel plugs were treated with freshly prepared 10 mM DTT in 50 mM NH_4_HCO_3 _for 45 min at 56°C. After incubation, the DTT was replaced quickly by the same volume of freshly prepared 55 mM iodoacetamide in 50 mM NH_4_HCO_3 _for 30 min and then dehydrated with 100% ACN. The dried gel pieces were incubated for 12 h at 37°C with 25 mM NH_4_HCO_3 _containing 0.02 μg/μl of mass spectrometry grade trypsin (Promega, Madison, WI, USA). The resulting peptides were extracted twice from the gel pieces, using peptide extraction buffer (1:1 mixture of 70% ACN and 0.1% trifluoroacetic acid [TFA]).

### Mass spectrometry

Mass spectrometry was carried out as described earlier [34]. Digested samples were desalted and concentrated on C-18 ZipTips (Millipore, Billerica, MA, USA) using the manufacturer's protocol. ZipTips were eluted on MTP 384 target plate with 2 μl of α-cyano-4-hydroxycinnamic acid (HCCA) (Sigma-Aldrich, USA) saturated solution dissolved in 50% ACN, 0.2% TFA. Mass spectra of digested proteins were acquired using Autoflex II TOF/TOF 50 (Bruker Daltonik GmbH, Leipzig, Germany) in positive reflectron mode, in the detection range of 500-3000 m/z. External calibration to a spectrum, acquired for a mixture of peptides with masses ranging from 1046 to 2465 Da, was done prior to acquisition. The proteolytic masses obtained were then processed through Flex Analysis v.2.4 programme for peak detection. Submission of peak lists to the UniProtKB/Swiss-Prot database using the Mascot search engine http://www.matrixscience.com to identify the proteins from the annotated *M. tuberculosis *chromosome (strain H37Rv, EMBL/GenBank/DDBJ entry AL123456) Release 20 (June 2010). The pI and molecular mass of proteins were taken into account for identification of proteins and we did not find any significant difference between the experimental and predicted pI and molecular mass of proteins. Peptide mass tolerance was set to 50 ppm with carbamidomethyl-cystein set as fixed modification, oxidation of methionine as variable modification and 1 missed cleavage site allowed. Few intense peaks from each spectrum were selected for fragmentation by laser-induced dissociation in MALDI-TOF/TOF. The MS⁄MS spectra were calibrated internally to the precursor ion mass and used for sequence specific search at mascot database (Matrix science). In addition, peptide mass fingerprint-based searches were carried out using only the set of peptide masses, in the same database without any constraints for isoelectric point (pI) and molecular mass. The whole procedure was repeated several times to ensure correct protein identification.

### Bioinformatic analysis

Protein sequences of all nine overexpressed proteins were retrieved from Tuberculist server http://genolist.pasteur.fr/TubercuList/ hosted by Pasteur Institute, Paris for whole annotated genome of H37Rv. BLAST [37] runs were performed at NCBI server http://blast.ncbi.nlm.nih.gov/ using the default threshold E-value of 10 and inclusion threshold value of 0.005. Motif and domain searches were made on EBI server http://www.ebi.ac.uk/Tools/InterProScan/ employing InterProScan which uses 13 different homology search programs (blastprodom, fprintscan, pfam, pir, panther, tigr, smart, superfamily, gene3 d, scanregexp, profilescan, seg, coils, tm, signalp, GO). Orthologs of proteins from other species of mycobacteria and human were obtained from KEGG http://www.genome.jp/kegg/ by single-directional best-hit option (SBH) and same server was employed for multiple sequence alignments (CLUSTALW) [38] we have used the following combined set of 5 organisms: mtu (*M.tuberculosis*), mbo (*M.bovis*), mav (*M.avium*), mle (*M.leprae*) and hsa (*Homo sapiens*). Sequences of H37Rv were submitted for 3-dimentional structure predictions at I-TASSER server http://zhang.bioinformatics.ku.edu/I-TASSER/. Structures were selected on the basis of RMSD values and agreement with Ramachandran Plot using VMD software (University of Illinois). Selected structures were molecularly docked with SM (structure obtained from http://www.drugbank.ca for *in-silico *interactions studies by submitting the structures to Patch Dock server [39]http://bioinfo3d.cs.tau.ac.il/PatchDock/ which is based on shape complementarity principles and results were refined using FireDock server [40,41]http://bioinfo3d.cs.tau.ac.il/FireDock/ which rearranges the interface side chains and adjusts the relative orientation of the molecules. Interacting amino acid side chains, drug molecule orientation and docking feasibility was based on Fire Dock scores and visualizations with VMD software.

## Competing interests

The authors declare that they have no competing interests.

## Authors' contributions

PS carried out the experiments, participated in the data analysis and drafted the manuscript. BK helped in carrying out mass spectrometric experiments, YG participated in bioinformatic analysis and NS participated in 2DE experiments. VMK and KV helped in the design of the project as well as preparation and critical review of the manuscript. DB conceived and designed the study, interpreted the experiment data and drafted the manuscript. All authors read and approved the final manuscript.

## Supplementary Material

Additional file 1 **2DE patterns of three *M. tuberculosis *clinical isolates**. a, b & c are sensitive to all first line drugs. 500 μg of proteins were first separated on 17 cm IPG strips of pH 4-7 by IEF and then by 12% SDS-PAGE in second dimension. Proteins were stained by coomassie brilliant blue. Regions showing low expressed proteins are highlighted by circles and squares. Click here for file

Additional file 2 **2DE patterns of six *M. tuberculosis *clinical isolates**. a, b, c, d, e & f are resistant to SM. Regions showing overexpressed proteins are highlighted by circles and squares. Click here for file
