# Supplementary figures and images for: Proteomic analysis of streptomycin resistant and sensitive clinical isolates of Mycobacterium tuberculosis
Source: Proteome Sci. 2010 Nov 18;8:59. doi: 10.1186/1477-5956-8-59 (PMC2998474; doi:10.1186/1477-5956-8-59)

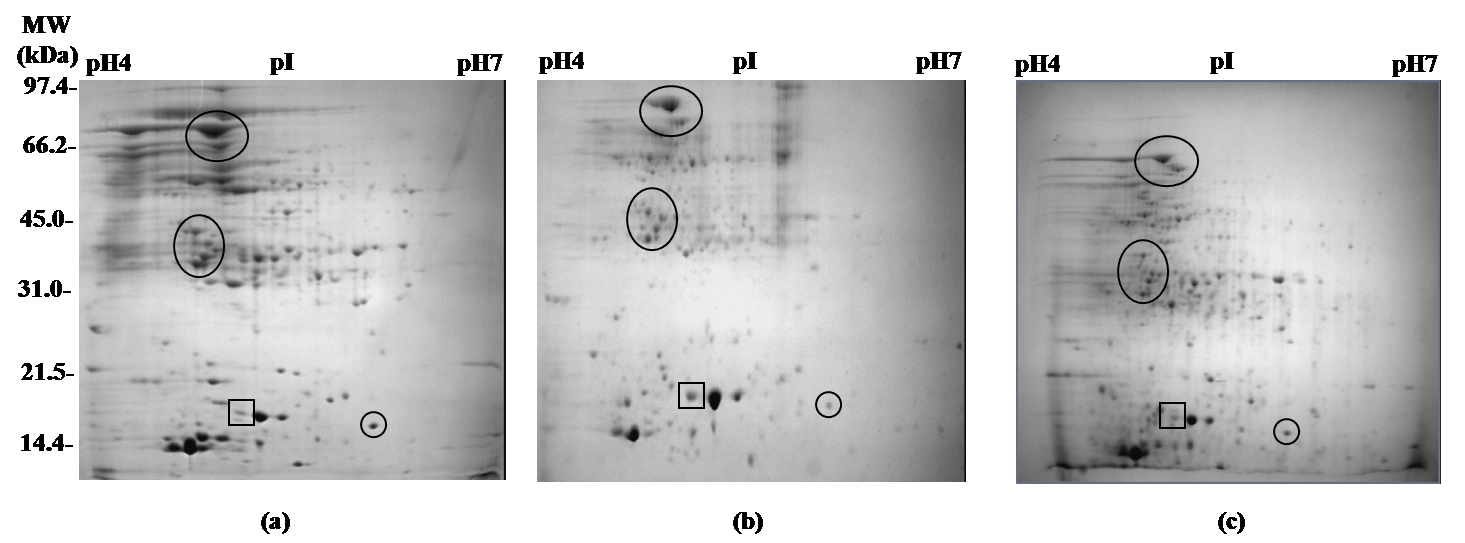

Supplement: Additional file 1 — 2DE patterns of three M. tuberculosis clinical isolates. a, b & c are sensitive to all first line drugs. 500 μg of proteins were first separated on 17 cm IPG strips of pH 4-7 by IEF and then by 12% SDS-PAGE in second dimension. Proteins were stained by coomassie brilliant blue. Regions showing low expressed proteins are highlighted by circles and squares. [file 1477-5956-8-59-S1.TIFF]

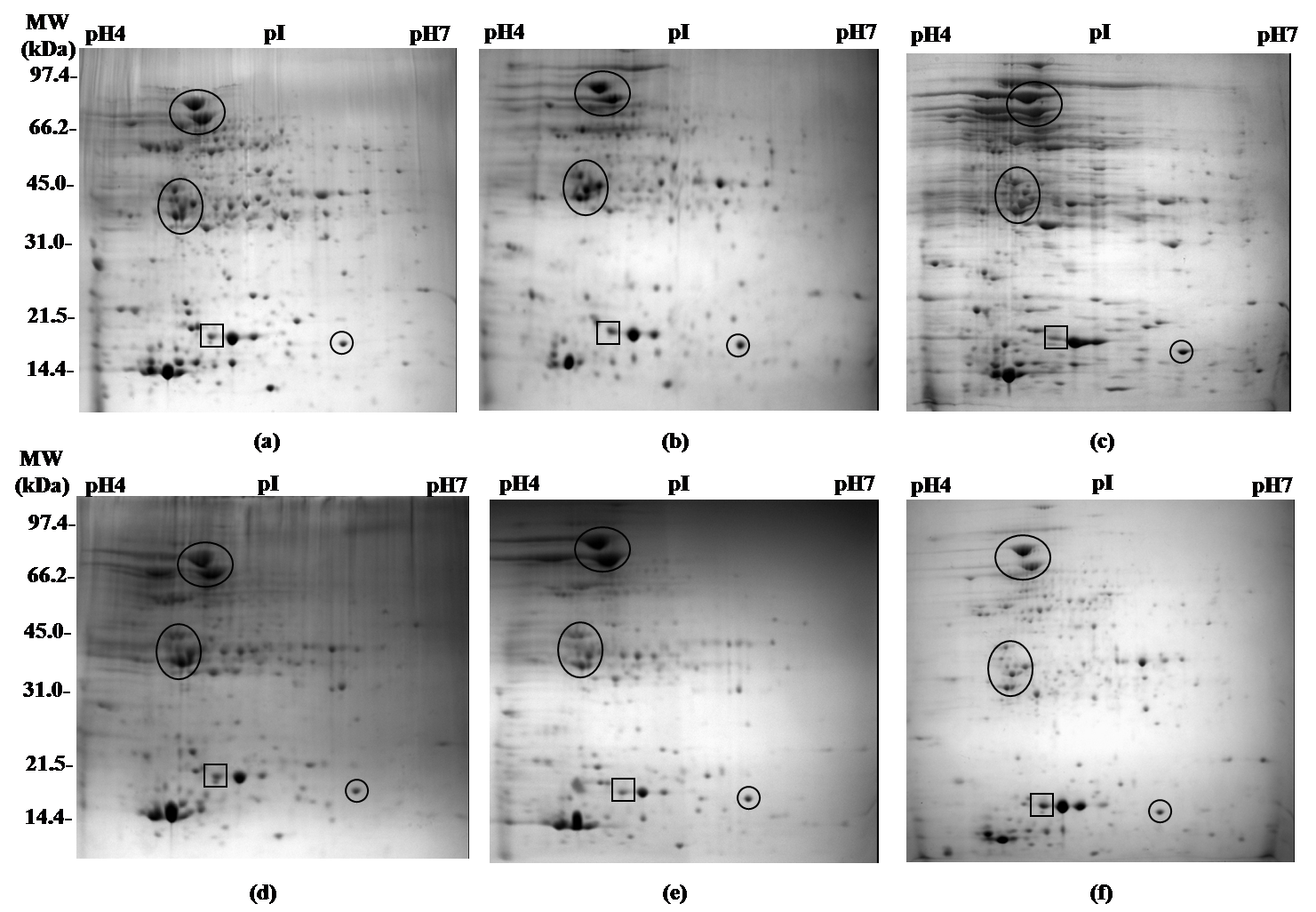

Supplement: Additional file 2 — 2DE patterns of six M. tuberculosis clinical isolates. a, b, c, d, e & f are resistant to SM. Regions showing overexpressed proteins are highlighted by circles and squares. [file 1477-5956-8-59-S2.TIFF]
